# United States Army Veterinary Service

**Published:** 1892-03

**Authors:** 


### EDITORIAL.

### UNITED STATES ARMY VETERINARY SERVICE.

A half loaf is better than none.—The several attempts to secure for the United States Army such a veterinary service as it should have, have proved futile for several reasons, the most serious of which being, we regret to acknowledge, because the American public is not yet sufficiently educated to appreciate the value of sound scientific basis for their guidance in every day life, and, with the generous resources of the country, making fortunes for the industrious, honest man and speculative charletan alike—it classes them alike—until its pockets are directly infringed upon. If we should have a war, and require the use of 50,000 or 100,000 horses, the authorities would establish a thorough veterinary service at once ; as we are at peace with the world and ourselves, and have but some 12,000 army horses, any service is considered sufficient.

A small factor in retarding the work for a veterinary service was the opposition of one or two prominent political men representing (?) the interests of the country in Congress, engendered by their relationship or personal association with quacks then holding positions as veterinary surgeons in the army, who feared any legislation which would demand an examination or investigation as to their competency. No systematic effort has been made to secure legislation for the improvement of the Army Veterinary Service in the present Congress, except one by the Army Veterinary Surgeons themselves, who reasonably ask for an increase in their present despicable pay. $75 a month—even without considering the high expenses of army posts, is absurd remuneration for men, who should have passed several years in obtaining their professional education, and whose profession requires a high order of mental ability and necessitates considerable physical hardship and danger.

A committee appointed by the Army Veterinary Surgeons from among themselves has had the following bill presented in Congress :

*S. 89. Mr. Cullom, Ill.*

*S. 885. Mr. Kyle, S. D.* A BILL

TO FIX THE PAY AND ALLOWANCES OF THE VETERINARIANS OF THE ARMY OF THE UNITED STATES.

*Be it enacted by the Senate and House of Representatives of the United States of America, in Congress assembled :*

SECTION 1. That the pay of the Veterinarians of the Army of the United States shall be One Hundred and Twenty-five Dollars

($125.00) per month with all the allowances of a Second Lieutenant of Cavalry.

SECTION 2. That the number of Veterinarians in the Army of the United States shall not exceed two (2) for each regiment of Cavalry.

SECTION 3. That hereafter all appointments as Veterinarians in the Army of the United States shall be confined to graduates of recognized Veterinary Colleges of the United States, and candidates for such appointments shall be citizens of the United States, and shall be required to pass such examination as the Secretary of War shall direct.

SECTION 4. That all Veterinarians—employed as such—in the Army of the United States at the passage of this Act shall be immediately reappointed without examination under the provisions of this Act.

SECTION 5. This Act to take effect immediately.

This bill is reasonable in all points, except the contradiction in Section 3, which says, "that hereafter all appointments . . . shall be confined to graduates . . . etc., and in Section 4, says, "that *all* veterinarians—employed as such— . . . shall be immediately reappointed . . . etc.

This bill has been favored by all veterinarians of the service, save one, and is heartily endorsed, both by Secretary Elkins and General Schofield, in fact, it was drawn up under the personal supervision of the latter.

It has been favorably recommended by the sub-committee of the military committee, having it in charge in the Senate, and no doubt it will be favorably endorsed by the entire committee. It has also been recommended by the War Department. Now is the time for one supreme effort in behalf of Army Veterinarians, and in fact of the veterinary profession of the country.

In the passage of this bill we will be starting at the small end of the wedge, and other improvements will follow. Cavalry officers of high rank in four cavalry regiments are unanimous that veterians should be commissioned officers, yet they cannot tolerate empirics as officers, and this is the consensus of opinion of most officers.

General Schofield said, "the veterinarians of the army can never expect anything worth having as long as there are 'quacks' in the service."

But what is to be done? two men who have served over thirty years in the Army, though empirics, are supposed to deserve some consideration, as these two men are considered invaluable in their regiments, not for their professional ability, but for certain work that can be carried on "between days." One of them is a canteen bartender, yet any attempt to remove him, save by retirement, would meet with the greatest opposition in his regiment.

Small matters like these will, however, right themselves, and we believe the passage of the above bill will do good as a stepping stone to something better.

As our self-sufficient colleagues in the Army feel that they are a band that must live or die—the good and the bad—together, and have not considered it worth while to call upon the United States Veterinary Medical Association for aid, nor to make their wants known to the veterinary profession at large, through the pages of the JOURNAL, which has always been willing to go to any trouble or expense for them, we cannot give further details, or advise our readers as to whom in the Army they can address. The JOURNAL will, however, gladly publish matter in regard to what can be done for the passage of this bill, and urges upon its readers to communicate at once with their representatives and senators, urging them to assist in its passage, and requesting answers from them, so that we can know where special work should be done.

